# Combined molecular dynamics and continuum solvent studies of the pre-pore Cry4Aa trimer suggest its stability in solution and how it may form pore

**DOI:** 10.1186/1757-5036-3-10

**Published:** 2010-05-13

**Authors:** Taveechai Taveecharoenkool, Chanan Angsuthanasombat, Chalermpol Kanchanawarin

**Affiliations:** 1 Department of Immunology, Faculty of Medicine, Siriraj Hospital, Mahidol University, Bangkok 10700, Thailand; 2 Laboratory of Molecular Biophysics and Structural Biochemistry, Institute of Molecular Biosciences, Mahidol University, Salaya Campus, Nakornpathom 73170, Thailand; 3 Theoretical and Computational Biophysics Laboratory, Department of Physics, Faculty of Science, Kasetsart University, Bangkok 10900, Thailand

## Abstract

Cry4Aa toxin is one of the highly specific mosquito-larvicidal proteins produced by the bacterium *Bacillus thuringiensis *subspecies *israelensis*. It is thought to form pores in the larval midgut membrane that cause membrane leakage and subsequent insect death. Therefore, Cry4Aa and other Cry toxins have been used as efficient and safe bacterial insecticides to control the disease-carrying mosquitoes such as *Aedes*, *Anopheles*, and *Culex*. However, we still do not clearly understand how Cry toxins kill mosquito-larvae at molecular details. Recent electron crystallographic images of Cry4Ba toxin, another toxin closely related to Cry4Aa toxin, have suggested that the protein forms trimer in aqueous solution and in lipid monolayer. Moreover, the unit cell of X-ray crystal structure of Cry4Ba toxin has been shown to be trimeric. In this study, we constructed the first full-atom structural model of Cry4Aa trimer using the trimeric unit cell structure of Cry4Ba toxin as a template and then used the methods of molecular dynamics (MD) and molecular mechanics combined with Poisson-Boltzmann and surface area (MM-PBSA) to show that the trimeric structure of Cry4Aa toxin is stable in 150 mM KCl solution on 10 ns timescale. The results reveal that Cry4Aa toxins use polar amino acid residues on *α*-helices 3, 4, and 6 to form trimer and suggest that the proteins form trimer to reduce their non-polar interactions with surrounding water. Based on the obtained trimeric structure of Cry4Aa toxins, we propose that pore formation of Cry toxins may involve a 90°-hairpin rotation during the insertion of their three *α*4-*α*5 hairpins into the membrane. This process may be mediated by water and ions.

**PACS Codes**: 87.15.ap, 87.15.bk, 87.14.ep

## 1. Introduction

Cry4Aa is a highly specific mosquito-larvicidal protein produced by the Gram-positive bacterium *Bacillus thuringiensis *subspecies *israelensis *(*Bti*). It is one of several toxins produced by the bacterium in the form of crystalline inclusions during its sporulation phase [1]. These cytoplasmic inclusions consist of at least four types of mosquito-larvicidal proteins (Cry4Aa, Cry4Ba, Cry11Aa and Cyt1Aa) which can be classified into two families known as crystal (Cry) and cytolytic (Cyt) *δ*-endotoxins [2,3]. In particular, the *Bti *Cry toxins have been used as efficient and safe bacterial insecticides to control the disease-carrying mosquitoes such as *Aedes*, *Anopheles*, and *Culex *[1]. However, we still do not clearly understand how Cry toxins kill mosquito-larvae at molecular details.

Pore formation is thought to be one of the molecular mechanisms for larvicidal activity of the *Bti *Cry toxins [3]. It can be considered as a series of important steps, including proteolytic activation, toxin oligomerization, and membrane insertion. The activation occurs when the toxin inclusions are solubilized by the highly alkaline solution in the midgut lumen. This releases soluble protoxins which are then digested by larval midgut proteases to form 65-kDa active toxins. During the oligomerizaion step, the monomeric active toxins have been suggested to bind together to form pre-pore trimers in solution [4] prior to binding to specific receptors located on the apical brush-border membrane of midgut epithelium. Subsequent conformational changes allow the insertion of their pore-forming portions into the cell membrane to form pores [5]. These pores are permeable to ions and small solutes causing membrane leakage that leads to osmotic lysis of midgut cells and finally insect death.

Structural data from X-ray crystallography of six different *Bt *Cry toxins, Cry1Aa [6], Cry2Aa [7], Cry3Aa [8], Cry3Ba [9], Cry4Aa [10], and Cry4Ba [11], reveal high degree of overall structural similarity. These proteins contain three distinct domains. The N-terminal domain I is a bundle of seven *α*-helices in which the *α*5 is surrounded by other helices. This domain has been shown to be responsible for pre-pore oligomerization [12-14], membrane insertion and pore formation [15,16]. Domain II is composed of three antiparallel *β*-sheets with highly varying amino acid sequence at the surface-exposed loops. This domain has been shown to participate and determine the specificity of the toxin for target insect larvae [17,18]. The C-terminal domain III contains a sandwich of two antiparallel *β*-sheets. This domain has been implicated in insect specificity [19-21] or ion regulation [22].

Recent structural studies of Cry toxins have allowed us to construct a full-atom model of pre-pore Cry4Aa trimer. Several evidence has suggested that Cry toxins may form pre-pore in solution before inserting into the membrane [4,12-14]. Electron crystallographic studies have also shown that the pre-pore structure of Cry4Ba toxin, another toxin closely related to Cry4Aa toxin, is trimeric in solution [4] and in lipid monolayer [4,5]. Moreover, a unit-cell structure of Cry4Ba toxin solved by X-ray crystallography has been shown to have a three-fold symmetry [11] suggesting that the trimeric coordinate of Cry4Ba toxin could be used as a full-atom model of a Cry4Ba pre-pore trimer in MD studies. However, the X-ray structure displays a missing part comprising helices *α*1 and *α*2 of domain I [11]. One possible alternative is to use the monomeric structure of Cry4Aa toxin which has been fully solved with all parts including *α*1 and *α*2 [10]. In addition, structure and sequence alignments between Cry4Aa and Cry4Ba showed high similarity in sequences (55% similarity) [23] as well as structures (RMSD 2.5 Å). Therefore, we decided to build a full-atom model of pre-pore Cry4Aa trimer.

Is this pre-pore trimeric structure of Cry toxins stable in an aqueous solution on a molecular dynamics (MD) simulation timescale? To address this question, we set up atomic-scale MD simulations and used molecular mechanics combined with Poisson-Boltzmann and surface area calculations (MM-PBSA) to investigate the structural stability of the pre-pore Cry4Aa trimer in 150 mM KCl solution on 10 ns timescale by comparing it energetically with a 10-ns MD simulation of Cry4Aa monomer in the same solution. This allowed us to observe how amino acid residues at the inter-subunit interfaces of the pre-pore trimer adjusted during course of the simulations as well as to study their key interactions. A conceivable notion for membrane insertion and pore formation by this toxin was also proposed. The results of this work would be paving the way for further MD simulations and mutagenic studies to gain more insights into the toxicity mechanism of the Cry toxins, particularly at the toxin oligomerization and pore formation steps.

## 2. Methods

### 2.1 Preparation of pre-pore Cry4Aa trimer and Cry4Aa monomer in KCl solution

The crystal structure of the 65-kDa activated Cry4Aa toxin was used to construct a structural model of pre-pore Cry4Aa trimer with its atomic coordinates taken from the Protein Data Bank (PDB) with PDB code 2C9K[10]. The structure consists of 598 amino acid residues, 252 water molecules and one methyl-2,4-pentanediol molecule which was then removed. The amino acid residues start from residues 68 to 679 with 14 missing residues between residues 235 and 244 (10 residues); and between residues 485 and 488 (4 residues). These missing residues correspond to two loops connecting *α*5 and *α*6; and *β*9 and *β*10, respectively. The two missing loops were modeled and incorporated into the structure using Loopy program [24,25] with the first loop having Arg^235 ^replaced by a glutamine to reproduce the mutant used in the X-ray crystallographic experiment [10].

Since there is no trimeric structure of Cry4Aa toxin available, a structural model of pre-pore Cry4Aa trimer was constructed by performing structure alignment using the incomplete trimeric structure of Cry4Ba from PDB code 1W99 as a template [11]. The Cry4Aa trimeric structure was obtained by aligning *α*3, *α*4 and *α*5 of the Cry4Aa monomeric structure with that of the Cry4Ba trimeric structure using DaliLite v.3 program [26]. Only *α*3, *α*4 and *α*5 were used for structure alignment because they are at the contact interfaces between Cry4Aa monomers.

A full-atom system of Cry4Aa trimer in a 150 mM KCl solution box was constructed using several molecular builder tools in VMD program [27]. A protein structure file (PSF) of Cry4Aa trimer was generated using Auto PSF Builder Tool with CHARMM27 force field [28]. All ionizable amino acid residues such as Arg, Lys, Asp and Glu were assigned to be in their charged states corresponding to an alkaline condition with pH 9.0. Then the Cry4Aa trimer was solvated in a water box with 12 to 15 Å buffering distance. Finally, 94 potassium (K^+^) and 88 chloride (Cl^-^) ions were placed randomly into the system to make 150 mM KCl solution. All K^+ ^and Cl^- ^ions were placed at distances more than 5 Å away from the proteins. The final system of the modeled Cry4Aa trimer has 223,439 atoms consisting of three 65-kDa Cry4Aa proteins (3 × 9,771 atoms), 64,648 water molecules, 94 K^+ ^ions, and 88 Cl^- ^ions, and has a dimension of 150 × 150 × 105 Å^3^.

Similarly, an all-atom system of Cry4Aa monomer in a 150 mM KCl solution box was also constructed for energy comparison with the trimer system. A Cry4Aa monomer was solvated in a water box of size 92 × 102 × 78 Å^3^. Then 32 potassium and 30 chloride ions were added randomly around the protein. The final system has 76,712 atoms (about one third of the trimer system) and consists of one Cry4Aa toxin (9,771 atoms), 22,293 water molecules, 32 K^+ ^ions and 30 Cl^- ^ions.

### 2.2 MD simulations of pre-pore Cry4Aa trimer and Cry4Aa monomer in KCl solution

MD simulations of the modeled Cry4Aa trimer in KCl solution were performed using NAMD 2.6 program [29], CHARMM27 force field [28], and TIP3P model for water [30] on 96 CPU-cores of a 2.3 GHz AMD Opteron computer cluster at 2 days per ns. The system was energy minimized and then equilibrated for 10 ns at temperature 298 K and pressure 1 atm with all heavy atoms of the proteins under harmonic restraints before equilibration using a force constant of 2 kcal/mol/Å^2^. The simulations were performed using periodic boundary conditions. Temperature and pressure were controlled using Langevin temperature control with damping constant of 5 ps^-1 ^and Langevin piston control [31] with an oscillation period of 200 fs and a damping time of 100 fs. An integration time step of 1.0 fs was used to allow a multiple time-stepping algorithm [32,33] to be employed such that long-range electrostatic interactions were computed every 4 time steps using the particle mesh Ewald method [34,35] with a grid spacing of ~1 Å for the reciprocal sum. van der Waals interactions were calculated every step using a 13 Å cutoff and a switching function. MD simulations of the Cry4Aa monomer system were performed in the same way as that of the trimer using the same simulation conditions on 32 CPU-cores of a 2.3 GHz AMD Opteron computer cluster at about 2 days per ns. From the simulations, both Cry4Aa trimer and monomer systems reached equilibrium within the first 5 ns and were very stable during the 10-ns equilibration.

### 2.3 Binding free energy calculations of the pre-pore Cry4Aa trimer in continuum solvent

To show that the pre-pore Cry4Aa trimer in solution is more stable than three isolated Cry4Aa monomers in solution, the method of molecular mechanics combined with Poisson-Boltzmann and surface area (MM-PBSA) calculations [36,37] was used to calculate the binding free energies of three Cry4Aa monomers in Cry4Aa trimer. Fifty snapshots of protein structures were taken at 100 ps interval from the last 5 ns of the two MD trajectories of Cry4Aa trimer and Cry4Aa monomer. For each snapshot, protein coordinate and structure files in AMBER file format were prepared for MM-PBSA calculations using AMBER force fields [38]. Then MM-PBSA calculation was performed on each snapshot using AMBER 9 [39] and then energies obtained were averaged over the fifty snapshots.

The binding free energies Δ*G*_*bind *_of the pre-pore Cry4Aa trimer were calculated as the difference between the free energy of Cry4Aa trimer () and that of the three isolated Cry4Aa monomers () according to a simple thermodynamics cycle:(1)

where the free energies  and  were defined as the sum of the molecular mechanical energies *H*_*MM*_, the solvation free energies *G*_*sol *_and the entropic energies -*TS*_*tot*_:(2)

The molecular mechanical energy *H*_*MM *_was computed by summing five contributions, i.e. three bonded interactions due to bond stretching, bond bending and bond torsioning, and two non-bonded interactions due to electrostatic and van der Waals interactions, according to the following AMBER potential energy function [38](3)

The first term corresponds to bond stretching, where *k*_*b *_is a bond force constant and *b *- *b*_0 _is a bond extension from equilibrium. The second term is due to bond bending, where *k*_*θ *_is an anglular force constant and *θ *- *θ*_0 _is an angular deviation from equilibrium. The third term is for twisting of dihedral angles around bonds where *k*_*φ *_is a dihedral force constant, *n *is the multiplicity of the function, *φ *is a dihedral angle and *δ *is a phase shift. The fourth term accounts for the van der Waals (vdW) potential calculated using 12-6 Lennard-Jones potential. The fifth term corresponds to electrostatic potential between all (i, j) pairs of atoms in the system, where *ε *is the dielectric constant of the medium.

The solvation free energy *G*_*sol *_was calculated as the sum of its polar and non- polar contributions ( and ). The polar part  was computed by solving Poisson-Boltzmann equation of the proteins in a continuum solvent model that has a dielectric constant *ε *= 80. The non-polar part  due to cavity formation and van der Waals interactions between the protein and the solvent was estimated as a solvent-accessible surface area dependent term , where A is the solvent-accessible surface area of the protein.

The total entropic energy *TS*_*tot *_of the protein was determined as the sum of that due to translation (*TS*_*tran*_), rotation (*TS*_*rot*_), and vibration (*TS*_*vib*_) at room temperature *T *= 298K using the normal mode analysis module of AMBER program [39]. The energy *TS*_*vib *_of the protein due to vibrational entropy was obtained from the sum of the contributions from all normal mode frequencies *f*_*i*_, where *i *= 1, 2, 3, ..., 3N - 6:(4)

This calculation has been done only for Cry4Aa monomer (containing 9,771 atoms) but not for Cry4Aa trimer (containing 29,313 atoms) because the number of atoms in the trimer was too large for the normal mode analysis module of AMBER program to handle. However, other components of the total entropic energy of Cry4Aa trimer could be estimated using statistical mechanics and the entropic energies obtained from the Cry4Aa monomer. The translational and rotational entropies  and  of the trimer were estimated from(5)(6)

where  and  are the translational and rotational entropies of Cry4Aa monomer, *m*_1 _= 6.98 × 10^4 ^*amu *and *I*_1 _= 3.33 × 10^7 ^*amu*.Å^2 ^are the mass and the moment of inertia of a Cry4Aa monomer and *a *= 28.5Å is the distance between the center of mass of a Cry4Aa monomer and that of a Cry4Aa trimer. Since all the normal mode frequencies of the Cry4Aa trimer could not be obtained and MM-PBSA calculations of other proteins complexes seem to suggest that the vibrational entropic energy change  due to complex formation is of order a few kcal/mol [40,41], so the vibrational entropy  of the Cry4Aa trimer was approximated to be about three times that of Cry4Aa monomer.

## 3. Results and discussion

**Figure 1:**
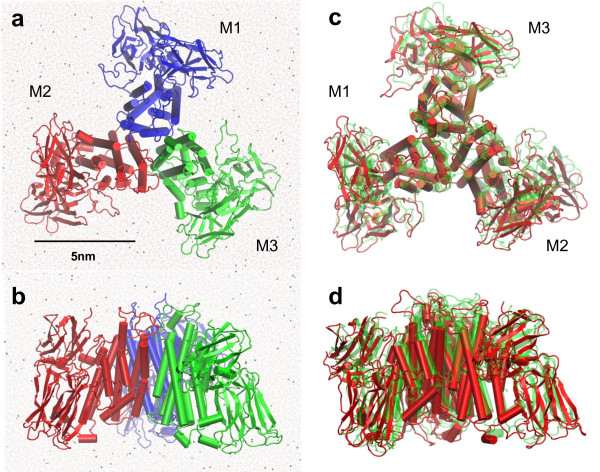
**The MD system of pre-pore Cry4Aa trimer in KCl solution**. (a) top view, and (b) side view of the system at time t = 10 ns. Three Cry4Aa monomers, i.e. M1, M2, and M3, are rendered in cartoon representation and colored blue, red and green, respectively. Oxygen of water molecules are rendered as red dots while K^+ ^and Cl^- ^ions are rendered as gold and blue spheres respectively. (c) top view, and (d) side view of Cry4Aa trimer at time t = 10 ns (red) compared with that at time t = 0 ns (transparent green). They are rotated by 60° about its symmetric axis.

The results from the MD simulations suggest that the trimeric structure of the pre-pore Cry4Aa toxin is stable in aqueous solution during 10 ns as shown in Figure 1a and 1b. Overall, the structure of Cry4Aa trimer did not change much from the initial structure with all three monomers still intact as can be seen from Figure 1c and 1d. This suggests that the interactions among the three Cry4Aa monomers and their surrounding water may be strong enough to bind them together in the solution on 10-ns timescale. So we investigated the structural changes and the binding between monomers by viewing the system, and calculating radii of gyration (R_gyr_), root mean square deviations (RMSD), root mean square fluctuations (RMSF) and binding free energy. Finally, we proposed how Cry toxins may form pore.

### 3.1 Radius of gyration and RMSD of C_*α *_atoms versus time

**Figure 2:**
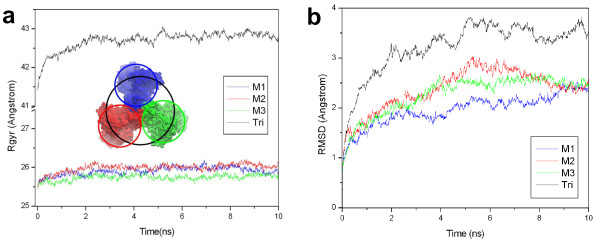
**Radius of gyration (R_gyr_) and root mean square displacement (RMSD)**. (a) R_gyr_, and (b) RMSD of C^*α *^atoms in pre-pore Cry4Aa trimer (Tri, colored black) and three monomers (M1, M2 and M3, colored blue, red, and green, respectively) during the 10-ns MD simulations. Each data point is calculated at 10 ps interval. Trimer in (a) is rendered as surface with circles drawn to show R_gyr_.

To measure the overall change of the trimeric structure of the pre-pore Cry4Aa, the radius of gyration (R_gyr_) and RMSD values of all C*_α_*atoms in the modeled trimer and its three monomers were computed. The R_gyr _plot in Figure 2a shows that the trimer has expanded radially from its center of mass by about 1.4 Å while the three monomers have expanded by about 0.5 Å from their centers of mass. The R_gyr _values of each monomer is about 1 Å less than that of the trimer because Cry4Aa trimer has a propeller-like shape which causes a small increase in R_gyr _of each monomer to result in a larger increase in R_gyr _of the trimer. The RMSD plot in Figure 2b also supports the 1-Å radial expansion of Cry4Aa trimer as it can be seen that the RMSD value for the trimer is about 1 Å larger than that of the monomer. Most of the changes in R_gyr _and RMSD values occur during the first 5 ns and after that the values become steady indicating that after 5 ns the system approaches equilibrium.

### 3.2 Structural fluctuation of C_*α *_atoms by residue

**Figure 3:**
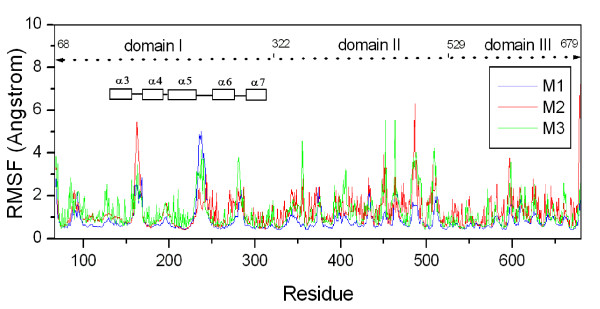
**Root mean square fluctuation (RMSF) by residue**. RMSF for the three Cry4Aa monomers M1 (blue), M2 (red) and M3 (green) in pre-pore Cry4Aa trimer from 10-ns MD simulations. *α*3, *α*4, *α*5, *α*6 and *α*7 in domain I are drawn as squares for indication of flexible loops.

To investigate the structural flexibility of the pre-pore Cry4Aa trimer, RMSF value was calculated for each monomer on Cry4Aa trimer as shown in Figure 3. Most of flexible parts of Cry4Aa can be seen as peaks in the plot, for examples, the loops connecting *α*3 and *α*4, *α*4 and *α*5, and *α*5 and *α*6 in domain I. The loop connecting *α*4 and *α*5, which has been shown to be critically involved in forming pore [15], has low fluctuation, indicating that this loop is more rigid than other loops in Cry4Aa trimer. This rigidity has been shown to be due to a disulphide bond (Cys^192^-Cys^199^) and a proline-rich motif (Pro^193^ProAsnPro^196^) within the loop [42].

### 3.3 Amino acid residues at the inter-subunit interfaces

**Figure 4:**
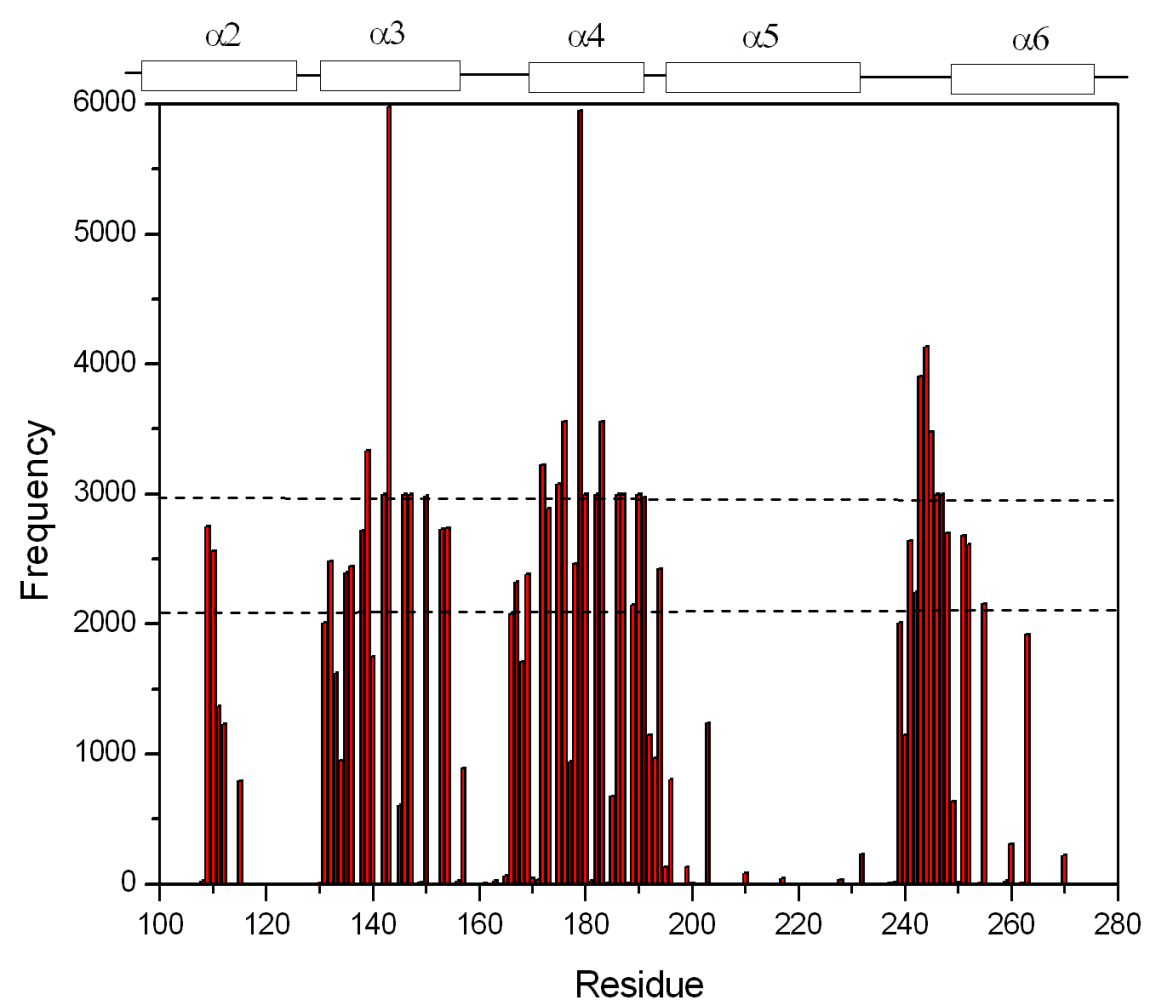
**Frequency distribution of amino acid residues at inter-subunit interfaces of the pre-pore Cry4Aa trimer**. Frequencies that amino acid residues appear within a distance of 3.5 Å from the inter-subunit interfaces of three Cry4Aa monomers in the pre-pore trimer during 10-ns MD simulations. The dotted line at frequency of 2,980 is used to indicate amino acid residues that are in contact 100% of the simulation time while the dotted line at 2,100 is for 70%.

To indentify amino acid residues that participate in binding between two Cry4Aa monomers in the pre-pore trimeric structure, we counted amino acid residues to see how often they were within a distance of 3.5 Å from the inter-subunit interfaces of each Cry4Aa monomer from 1,000 frames of the 10-ns MD trajectory (10 ps per frame). The results are presented as a histogram shown in Figure 4. It can be seen that most of amino acid residues at the inter-subunit interfaces are mainly residues on *α*3 and *α*4 and partially on *α*2, the loop connecting *α*5 and *α*6, and the N-terminal of *α*6.

**Figure 5:**
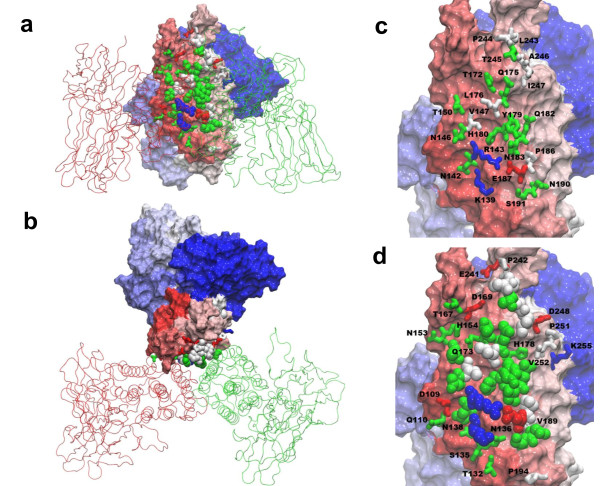
**Amino acid residues at the inter-subunit interface of a Cry4Aa monomer within the pre-pore trimer**. (a) side view with Group 1's residues rendered as van der Waals (vdW) spheres and Group 2's residues rendered as sticks. Green is for polar and white is for non-polar. Blue is for positively charged and red is for negatively charged. One of Cry4Aa is depicted as surface and colored in spectrum from red to white to blue by residue number. The other two Cry4Aa monomers M2 and M3 are rendered as lines and colored red and green. (b) top view of (a), (c) Group 1's residues rendered as sticks, and (d) Group 2's residues depicted as sticks with Group 1's residues depicted as vdW spheres.

From Figure 4, we have classified the amino acid residues that are within 3.5 Å distance from the inter-subunit interfaces of three Cry4Aa monomers into two groups. Group 1 consists of amino acid residues that are within the inter-subunit interfaces for almost 100% of the time or more with frequency more than 2,980 or more than ~1,000 from each interface (The frequencies of more than 3,000 come from some amino acid residues being at the interfaces near the center of the trimer, so they are counted more than once per interface). Group 2 consists of residues that are within the inter-subunit interfaces for more than 70% of the time with frequency more than 2,100 but less than 2,980. These two groups of amino acid are located on *α*2, *α*3, *α*4 and *α*6, and their vicinity as listed in Table 1. Residues in Group 1 are mainly at the center of the inter-subunit interface of Cry4Aa monomer while residues in Group 2 are on the peripheral of the inter-subunit interface as shown in Figure 5. It can be seen clearly that most of the amino acid residues at the inter-subunit interfaces of Cry4Aa monomers are polar residues (e.g. Tyr^179^, His^180^, Asn^183 ^and Ser^191^) with a few charged (Arg^143^, Glu^187 ^and Lys^139^) and non-polar residues (e.g. Val^147^, Leu^176^, and Pro^186^).

**Table 1:** Amino acid residues at the inter-subunit interfaces of the pre-pore Cry4Aa trimer

Percentage of time in contact	Helix *α*2 (res95-126) of monomer M_i_	Helix *α*3 (res130-157) of monomer M_i_	Helix *α*4 (res169-191) of monomer M_i+1_	Helix *α*6 (res249-276) of monomer M_i+1_
Group 1		Lys^139^	Thr^172^	Leu^243b^
~100% or more^a^		Asn^142^	Gln^175^	Pro^244b^
		Arg^143^	Leu^176^	Thr^245b^
		Asn^146^	Tyr^179^	Ala^246b^
		Val^147^	His^180^	Ile^247b^
		Thr^150^	Gln^182^	
			Asn^183^	
			Pro^186^	
			Glu^187^	
			Asn^190^	
			Ser^191^	

Group 2				
	Asp^109^	Thr^132^	Thr^167^	Glu^241b^
	Gln^110^	Ser^135^	Asp^169^	Pro^242b^
more than 70% but less than 100%		Asn^136^	Gln^173^	Asp^248b^
		Asn^138^	His^178^	Pro^251^
		Asn^153^	Val^189^	Val^252^
		His^154^	Pro^194 c^	Lys^255^

Two groups of amino acid residues located at the inter-subunit interface of Cry4Aa monomers M_i _and M_i+1 _in Cry4Aa trimer where i and i+1 run in cyclic of 1, 2, and 3. ^a^The percentage of more than 100% was due to some residues were counted more than once because they were within two or more inter-subunit interfaces of Cry4Aa trimer. ^b^Residues on the loop between *α*5 and *α*6. ^c^Residue on the loop between *α*4 and *α*5.

**Figure 6:**
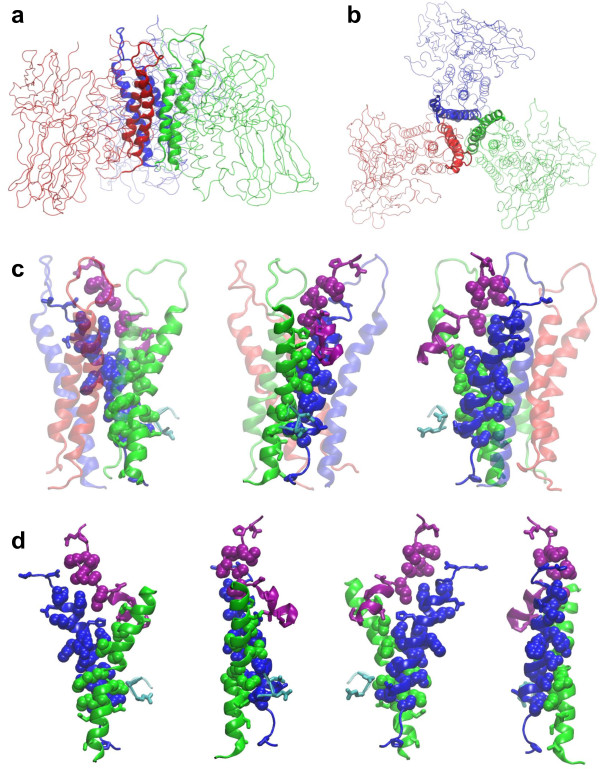
**One of three inter-subunit interface formed by *α*2 and *α*3 of a Cry4Aa monomer interlocking with *α*4 and *α*6 of an adjacent Cry4Aa monomer within the pre-pore trimer**. (a) side view (b) top view of three hairpins of *α*3 and *α*4 in Cry4Aa trimer. The hairpins are rendered as ribbons and colored blue for M1, red for M2 and green for M3, (c) three 120°-side views of amino acid residues at the interface between *α*2 and *α*3 of M3 (cyan and green) and helices *α*4 and *α*6 of M1 (red) with the three *α*3-*α*4 hairpins shown in transparent, and (d) Four 90°-side views of the interlocking between *α*3, *α*4 and *α*6 by Group 1's residues. Residues on *α*2 of M3 are colored cyan and that of *α*6 are colored purple. Residues of Groups 1 and 2 from Table 1 are depicted in vdW and stick representations, respectively.

By investigating the interfaces in details, it can be shown that the binding between two Cry4Aa monomers occurs specifically at the interfaces, for example, between *α*2 and *α*3 of one monomer (M3; in cyan and green), and *α*4, and *α*6 of adjacent monomer (M1; in blue and purple) as shown in Figure 6. The amino acid residues located at such interfaces are those listed earlier in Table 1 which dominantly are polar residues. In particular, Figure 6d shows that the polar residues from Group 1, such as Lys^139^, Asn^142^, Arg^143^, Asn^146 ^on *α*3, and Tyr^179^, His^180^, Gln^182^, Asn^183^, Glu^187^, Asn^190^, Ser^191 ^on *α*4, and Thr^245 ^on *α*6, are key residues that interlock with each other and form one of three inter-subunit interfaces within the Cry4Aa trimer.

### 3.4 Binding free energy of the pre-pore Cry4Aa trimer

From Table 2, the negative binding free energy (-100 kcal/mol) of the pre-pore Cry4Aa trimer in a continuum solvent obtained from the method of molecular mechanics combined with Poisson-Boltzmann and surface area (MM-PBSA) [36,37] suggests that Cry4Aa trimer is more stable than three separated Cry4Aa monomers in solution. The binding free energy is contributed negatively by the non-polar interacions between the proteins and water (-446 kcal/mol), the van der Waals interactions within the proteins (-10 kcal/mol), and possibly the vibrational entropy of the proteins. It is contributed positively by the polar interactions between the proteins and water (43 kcal/mol), the electrostatic interactions within the proteins (143 kcal/mol), the conformational changes of the proteins (105 kcal/mol) as well as the entropies due to translations and rotations of the proteins (34 and 36 kcal/mol). The large negative binding free energy contribution of -446 kcal/mol due to the non-polar interactions between the proteins and water possibly came from the formation of the inter-subunit interfaces between the three Cry4Aa monomers during their trimer formation. This removed water molecules from the inter-subunit interfaces of the three monomers and therefore decreased their water accessible surface area.

**Table 2:** Binding free energy of pre-pore Cry4Aa trimer in solution

Contributions	Trimer	**3 **× **Monomer**	Difference	Notes
**1**. ***G*_*sol*_**	-11,722.5^a ^± 17.6^b^	-11,319.6 ± 37.9	-402.9 ± 42.8	Solvation free energy

1-1.	0.9 ± 0.0	446.6 ± 1.0	-445.7 ± 1.0	Non-polar part
1-2.	-11,723.5 ± 17.6	-11,766.2 ± 37.9	42.8 ± 41.8	Polar part

**2**. ***H*_*MM*_**	-36,968.6 ± 22.1	-37,206.5 ± 46.5	237.9 ± 51.4	Molecular mechanical energy

2-1. *H*_*elec*_	-50,075.3 ± 20.2	-50,218.7 ± 43.7	143.4 ± 48.1	Electrostatic energy
2-2. *H*_*vdW*_	-9,430.9 ± 5.8	-9,420.8 ± 13.7	-10.2 ± 14.9	van der Waals energy
2-3. *H*_*conf*_	22,537.6 ± 6.6	22,432.9 ± 7.5	104.7 ± 10.0	Conformational energy

**3. -*TS*_*tot*_**	[~-19,430]^c^	-19,488.5	[~60]	Entropic energy
3-1.-*TS*_*tran*_	(-18.6)^d^	-52.9	(34.4)	Translational part
3-2. *TS*_*rot*_	(-20.0)	-56.3	(36.3)	Rotational part
3-3. *TS*_*vib*_	[**~**-19,390]	-19,379.3	[**~ **-10]	Vibrational part

**4**. ***G*_*tot*_**	-48,691.1 ± 28.2^e^	-48,526.1 ± 60.0	-165.0 ± 66.3	Free energy (1+2)
			[~-100]	Free energy (1+2+3)

Binding free energy (in kcal/mol) calculated as the difference between the free energy of Cry4Aa trimer in solution and that of three separated Cry4Aa monomers in solution.

^a ^Energy was calculated as an average over 50 snapshots taken at 100 ps interval from the last 5 ns of the 10-ns MD simulations.

^b ^Error in energy was calculated as the standard error of the mean.

^c ^Energies in square brackets were guessed values based on data from simulations of protein complexes [45,46].

^d ^Energies in round brackets were theoretical estimated values using statistical mechanics and the entropic energies of Cry4Aa monomer.

^e ^Error in adding or subtracting two values of energies *E*_1 _± Δ*E*_1 _and *E*_2 _± Δ*E*_2 _was estimated as .

On the other hand, the large positive binding free energy of 143 kcal/mol caused by electrostatic interactions within the proteins possibly arose from the net charge of -2 on each Cry4Aa monomer which may cause the increase in the free energy after trimer formation. Another 105 kcal/mol contribution of binding free energy probably resulted from the conformational stress within the proteins due to the inter-subunit interfaces of Cry4Aa trimer. Moreover, the binding free energy of 43 kcal/mol due to polar-solvation energy was likely contributed by the removal of water molecules from the inter-subunit interfaces as this decreased the electrostatic attractions between the proteins and water. However, further investigation using a combination of MD simulations of Cry4Aa mutants and MM-PSBA may be needed to identify key residues at the interfaces that mainly contribute to the negative binding free energy.

### 3.5 Proposal of a pore-forming mechanism for Cry toxins

**Figure 7:**
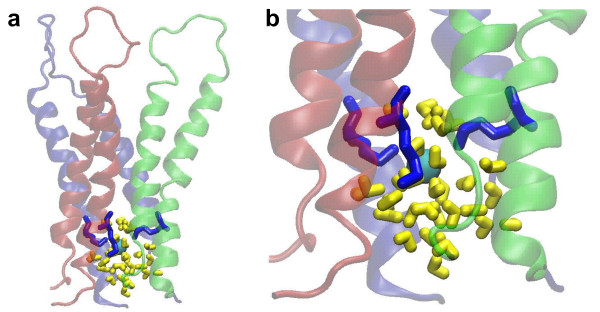
**Chloride ion trapped by three Lys^139 ^within a large water cavity at the bottom center of the pre-pore Cry4Aa trimer**. (a) side view, and (b) magnified view of three *α*3-*α*4 hairpins showing the water cavity in Cry4Aa trimer. The three hairpins are depicted as ribbons and colored blue, red, and green. Chloride ion is rendered as a cyan sphere. Three Lys^139 ^and water are rendered as blue and yellow sticks, respectively.

Why Cry toxins utilize hydrophilic core to form trimer and reduce their non-polar interactions with water? It has been shown that globular proteins can utilize a various means of protein interaction to form oligomers such as dimers [43]. One is to have a hydrophobic core at their interface while another is to have the opposite which is a hydrophilic core like Cry4Aa as observed here. Other different interaction may use a combination of both hydrophobic and hydrophilic patches at protein interface. In the case of Cry4Aa toxin, this may be related to their pore-forming function that requires to have polar environment for the pore interior. In this work, we have also observed a chloride ion being trapped by three Lys^139 ^in a large water cavity at the bottom of the interface within the Cry4Aa trimeric structure as shown in Figure 7. It is possible that, during pore formation, water molecules and ions are involved in breaking of some hydrogen bonds, and salt bridges, and overcoming steric interactions between helices in domain I of Cry toxins so that *α*4 and *α*5 can be inserted into the membrane bilayer and rearranged to form pore.

**Figure 8:**
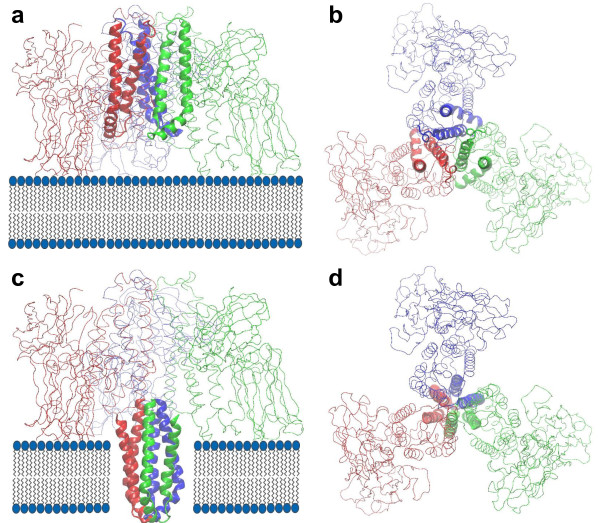
**Pore formation of the pre-pore Cry4Aa trimer by insertion of three *α*4-*α*5 hairpins into a membrane bilayer**. (a) side view, and (b) top view before insertion, (c) side view, and (d) top view after the insertion of the three hairpins with 90° hairpin rotation to form a barrel that has a channel at the center. The three hairpins are rendered as ribbons and colored blue for M1, red for M2, and green for M3. Other parts of the protein are drawn as lines.

Based on several experimental evidences and the obtained full-atom structural model of Cry4Aa trimer, we would like to propose a mechanism that suggests how Cry toxins may form pore. Recent electron crystallographic images of Cry4Ba toxin have shown that there is a pore at the center of Cry4Ba trimer [5]. These pores formed by Cry4Ba toxins can release calcein molecules trapped in lipid vesicles [4]. The pore diameters of Cry4Ba and Cry1C toxins [4,44] have been estimated to be about 20-26 Å which is about the diameter of an alpha helix (including sidechains). It has been shown that the pore of Cry4Ba toxin is formed by the insertion of *α*4-*α*5 hairpins into the membrane [45,46]. From our obtained structure of Cry4Aa trimer shown in Figure 8a, we think that the pre-pore Cry4Aa trimer has to be oriented relative to the membrane such that all three domain II of Cry4Aa trimer can interact with the receptors on the membrane surface [17,18] as well as all three *α*4-*α*5 hairpins are oriented in their inserting positions with their loops pointing toward the membrane. However, from the same figure if the three *α*4-*α*5 hairpins are inserted directly into the membrane by a distance of ~40 Å they cannot form pore and some reorientations of the three hairpins are required for all six helices to form a barrel. Therefore, during insertion the three hairpins in Figure 8a and 8b have to be rotated by about 90° clockwise so that all six helices can form a barrel that has a channel with the size of an alpha helix (about 15-20 Å) at the center as shown in Figure 8c and 8d. The opening and closing of the pore may be controlled by the tilting of the three *α*4-*α*5 helices relative to the normal to the membrane.

## 4. Conclusions

In this work, we have constructed the first all-atom structural model of the pre-pore Cry4Aa trimer based on the results from electron and X-ray crystallographic studies of Cry4Aa and Cry4Ba toxins. By using MD simulations, we have found that Cry4Aa toxin uses polar amino acid residues on helices *α*3, *α*4, and *α*6 to form trimer. A combination of MD and MM-PBSA calculations has suggested that Cry4Aa trimer is stable in aqueous solution mainly due to its formation of the inter-subunit interfaces which reduces the non-polar interactions between the proteins and their surrounding water. The results can later be investigated further by mutagenic studies to confirm the simulation results and help us understand more on the toxin oligomerization process. Moreover, we have proposed a pore-forming mechanism by suggesting that three *α*4-*α*5 hairpins are inserted into the membrane with each hairpin rotated by 90° clockwise (viewed from the top) to form pore during the insertion step of Cry toxins. This process may involve water molecules and ions.
